# Effects of uniconazole treatment on ‘Hass’ avocado productivity and gas-exchange parameters under Mediterranean climate

**DOI:** 10.3389/fpls.2025.1668625

**Published:** 2025-09-19

**Authors:** Michal Lahack, Nitzan Szenes, Lior Rubinovich

**Affiliations:** ^1^ Northern Agriculture R&D, MIGAL – Galilee Research Institute, Kiryat Shmona, Israel; ^2^ Avocado Professional Instructor (private), Ben-Ami, Israel; ^3^ Department of Biotechnology, Faculty of Science and Technology, Tel Hai College, Kiryat Shmona, Israel

**Keywords:** carbon assimilation, flowering, fruit weight, growth, *Persea americana*, yield

## Abstract

Avocado (*Persea americana* Mill.) productivity faces major challenges due to rising market demand, climate variability, excessive vegetative growth, and labor-intensive management practices. This study evaluated the effects of soil-applied uniconazole (UNI), a gibberellin-biosynthesis inhibitor, on vegetative development, flowering, gas exchange, and fruit yield in mature ‘Hass’ avocado trees. The experiment, conducted over 2 years in a commercial orchard in northeastern Israel, compared three UNI concentrations (8, 12, and 16 mL tree^-^¹) applied via drip irrigation in May 2022 and May 2024. UNI treatments significantly reduced trunk diameter and increased floral bud density, indicating effective suppression of growth. Flowering intensity was enhanced during the first season across all UNI treatments, but not in the following year. Chlorophyll content showed a significant increase only in May 2023 for the 12 and 16 mL tree^-^¹ treatments. Physiological measurements revealed increased carbon assimilation and stomatal conductance in UNI-treated trees, particularly during the first season. Despite reductions in total yield at higher UNI doses in the first year of the experiment, the 12 and 16 mL tree^-^¹ treatments produced significantly larger fruit with improved size-class distribution, offering potential market advantages. Overall, the 8 mL tree^-^¹ treatment provided the best compromise between suppressing vegetative growth and enhancing carbon assimilation without compromising yield. These findings underscore the potential of UNI for optimizing canopy architecture and fruit quality in avocado orchards, particularly under a Mediterranean climate. Long-term assessments are recommended to refine dose strategies and evaluate commercial viability.

## 1 Introduction

Avocado (*Persea americana* Mill.) is a high-value subtropical fruit crop cultivated extensively in regions with Mediterranean and tropical climates (Alcaraz et al., 2013). The black-skinned Guatemalan–Mexican hybrid avocado cultivar Hass holds significant commercial value, currently leading global avocado production (Schaffer et al., 2013). For instance, in 2018, ‘Hass’ accounted for approximately 97% of the total avocado market in the United States (Ballen et al., 2021), due to its market-preferred characteristics, including optimal fruit size, thick peel, buttery flavor, and smooth, creamy texture (Silva and Ledesma, 2014). In recent years, optimizing avocado productivity has become increasingly important due to rising market demand, climate variability, and labor-intensive management practices. Among the major challenges facing avocado production are excessive vegetative vigor and inefficient fruit set, which results from intense competition between flowers and developing fruitlets and the newly emerging vegetative shoots above the panicles (Symons and Wolstenholme, 1990; Lovatt and Salazar-Garcia, 2006). Controlling tree size is a significant challenge in the avocado industry. Avocado trees grow vigorously, which complicates pest management, harvesting, and other cultural practices, increasing labor and production costs. In spring, substantial energy reserves are directed toward vegetative shoot growth, potentially reducing the resources available for fruit development (Köhne and Kremer-Köhne, 1989). Avocado productivity is further limited by alternate bearing, defined as variation in yearly orchard yields (Ziv et al., 2014; Cohen et al., 2023). In recent years, mitigating the adverse effects of climate change has become a major concern for growers, driven by the rising frequency of extreme weather events (Zhang et al., 2012; Shapira et al., 2021; Lahak et al., 2024). These challenges negatively impact both yield stability and fruit quality.

Plant-growth regulators are among the most effective tools for controlling tree growth and enhancing yield, offering practical solutions to production challenges in established orchards. These compounds, when applied at appropriate concentrations and timings, can modulate the balance between vegetative and reproductive growth, reduce excessive shoot growth, enhance flowering, and improve fruit size and yield uniformity (Lovatt and Salazar-Garcia, 2006). Among the most commonly used plant-growth regulators in avocado are gibberellin (GA)-biosynthesis inhibitors, particularly the triazoles uniconazole (UNI) and paclobutrazol (PBZ) (Menzel and Le Lagadec, 2014; Brogio et al., 2018). These plant-growth retardants function primarily by inhibiting GA biosynthesis, resulting in reduced shoot elongation and potentially shifting resource allocation toward reproductive organs (Rademacher, 2000; Mitra, 2018). Numerous studies in ‘Hass’ avocado orchards have shown that applying these growth retardants during the flowering period by foliar spray can suppress vegetative growth, enhance flowering, and significantly improve fruit yield, shape, and size, thereby increasing growers’ economic returns (Adato, 1990; Wolstenholme et al., 1990; Erasmus and Brooks, 1998; Brogio et al., 2018). Another possible method of application is through the irrigation system, i.e., soil drenching. However, the appropriate concentrations for this method are still unclear; highly variable doses of UNI (as the active ingredient) have been used in past studies, ranging from 0.001 to 1 g L^-1^ (Bórquez-Lillo et al., 2015). This is a critical factor, particularly with irrigation-based applications, because the compound is delivered directly to the root zone and transported acropetally through the xylem (Davis et al., 1988). Moreover, due to the long-lasting persistence of these chemical agents in the soil, careful management of concentrations is essential to preventing prolonged suppression of tree growth (Menzel and Le Lagadec, 2014; Bórquez-Lillo et al., 2015).

The effect of the chemicals on fruit set and yield is less consistent than that on shoot growth; there have been many cases of the growth regulators decreasing shoot growth without any subsequent benefit in terms of productivity (Menzel and Le Lagadec, 2014). Moreover, the efficacy and long-term physiological consequences of such treatments in avocado remain inadequately understood, particularly under field conditions over multiple seasons. This study aimed to evaluate the effects of a UNI-based growth retardant applied by soil drenching via the irrigation system at increasing concentrations (8, 12, and 16 mL tree^-^¹) on vegetative growth, reproductive performance, gas-exchange characteristics, and fruit yield in mature avocado trees. UNI was selected because it is generally less persistent than PBZ, thereby avoiding long-term tree stunting. Specific objectives included (i) monitoring changes in trunk diameter, flowering intensity, and inflorescence bud density; (ii) assessing seasonal dynamics of photosynthesis, stomatal conductance, and chlorophyll content; and (iii) quantifying yield components and fruit-size distribution over two consecutive seasons. The findings provide insights into the potential of growth-retardant applications to enhance yield quality while managing vegetative vigor in avocado orchards.

## 2 Materials and methods

### 2.1 Experimental site

The experiment was conducted from May 2022 to February 2025 in a 0.8-ha commercial avocado orchard at Kibbutz Yiron in northeast Israel (33°09’N, 35°57’E, 92 m above sea level). The soil at the experimental location is classified by the USDA as clay, comprising 64.1% clay, 16.7% silt, and 19.2% sand, with a pH of 7.8. Extreme weather occurrences are highly likely at the trial site. Avocado cv. Hass grafted on seedling ‘Degania 117’ rootstock was planted in March 2019 with 3 m spacing between trees and 5 m between rows. The rows were oriented north/south. Trees were drip-irrigated at 9,000–9,500 m^3^ year^-1^ ha^-1^. Irrigation intervals were between 1 and 3 days and liquid fertilizer (NPK 8-1-8, Deshanim LTD., Israel) was supplied with each irrigation. `Ettinger` trees were planted every third tree in every third row as pollinizers.

### 2.2 Experimental design and uniconazole treatment

The experimental plot had a completely randomized design with four replicates (n = 4) for each treatment. Each replicate consisted of a row with 26 trees. Most measurements were taken only from the 8 middle trees in each row (experimental trees). UNI treatment was applied in May 2022 and May 2024 (once per year), coinciding with the post-harvest vegetative flush and before floral induction for the subsequent yield cycle in the experimental plot (Ziv et al., 2014). Magnum (50 g L^-1^ Uniconazole, Tapazol Ltd., Israel) was applied via the drip-irrigation system, which consists of an 18 mm diameter drip line, with each dripper having a flow rate of 1.6 liters per hour, and drippers spaced 50 cm apart. To ensure equal application per tree, Magnum was transferred into a fertigation tank, to which 50 liters of water were added, and irrigation was operated until all volume was applied. Subsequently, an additional 20 liters were added to the fertigation tank to wash any remaining material from the tank. Small valves were installed in the pipelines leading to each row in the orchard, so that during each Magnum application event, the valves corresponding to the specific rows of each treatment were opened separately. Care was taken to apply a fixed irrigation run-time. Final Magnum concentrations were 8-, 12-, and 16-mL tree^-1^, corresponding to 0.4, 0.6, and 0.8 g UNI tree^-1^, respectively. Data were gathered from the experimental plot from May 2023 to February 2025.

### 2.3 Meteorological measurements

The local `Kfar Blum` meteorological station provided data on precipitation and air temperature (Israeli Meteorological Services).

### 2.4 Tree trunk diameter, inflorescence bud density, and flowering-intensity estimation

The diameter of the tree trunks was measured using a Vernier caliper at the same height, after they had been marked 1–2 cm above the grafting point. During peak bloom in April 2023 and 2024, flowering intensity was assessed as previously described (Ziv et al., 2014; Bar-Noy et al., 2019). In a blind assessment, two independent surveyors evaluated each experimental tree individually on a scale of 0 to 5, where 0 represented no visible flowering and 5 indicated the highest flowering intensity. Measurements were taken from at least eight trees from each of the four replicates (n = 4). Inflorescence bud density (floral buds cm^-1^) was calculated by counting the number of floral buds along a 20-cm segment at the tip of a flowering branch and dividing that number by 20. Measurements were taken from at least three branches from the eight trees in each of the four replicates (n = 4).

### 2.5 Chlorophyll measurements

A chlorophyll meter (Apogee MC-100, Apogee Instruments, Logan, UT, USA) was used to measure the leaf chlorophyll concentration index (CCI). Measurements were taken from three leaves per tree, at least eight trees from each of the four replicates (n = 4).

### 2.6 Tree yield, average fruit weight, and size class distribution

On the orchard’s commercial harvest date in February 2024 and February 2025, fruit from the experimental trees in each replicate were manually picked and weighed separately for each tree. Average fruit weight (kg tree^-1^) was calculated by dividing the total fruit weight of each tree by the corresponding number of fruits on that tree. Measurements were taken from at least eight trees from each of the four replicates (n = 4). Commercial pack-out distribution by size grade class, expressed as the number of fruits required to fill a standard 4-kg carton (size class), was determined by categorizing and recording the number of fruits within predefined commercial weight classes for each tree (Garner et al., 2011; Micheletti et al., 2025). The size classes were based on the European classification for ‘Hass’ avocado and were as follows (grams per fruit): size 14 (266-305g), size 16 (236-265g), size 18 (211-235g), size 20 (191-210g), size 22 (171-190g), size 24 (156-170g), size 26 (146-155g), size 28 (136-145g), size 30 (125-135g) and 32 (80-125g). Measurements were taken from at least two trees from each of the four replicates (n = 4).

### 2.7 Leaf-level light intensity and gas-exchange measurements

A LI-6800 portable photosynthesis system (clear-top 6-cm^2^ chamber with a mounted small light source, LI-COR, Lincoln, NE, USA) was used to measure leaf-level photosynthetic photon flux density (PPFD), CO_2_-assimilation rate and transpiration rate. The airflow into the leaf chamber was approximately 700 μmol s^-1^, the CO_2_ fed into the chamber was set at 415 ppm, and the boundary-layer conductance to water vapor was approximately 3 mol m^-2^ s^-1^. The temperature and relative humidity in the chamber were set to be ambient. Mature attached leaves facing the sun were measured in the orchard at noon, and while the leaves were in the chamber, care was taken to keep them facing the sun in the same orientation. Selected leaves were taken from the southern side of the tree canopy, approximately 1.5 m above ground level, and positioned four to five leaves back from the branch tip. The LI-COR device calculated stomatal conductance to water vapor (g_s_). Leaf measurements were taken from three leaves per tree, at least two trees from the middle of each replicate (n = 4).

### 2.8 Statistical analysis

Data were analyzed using repeated-measures ANOVA (linear mixed model) in JMP version 18.2.2 (SAS Institute, Cary, NC, USA) to account for temporal dependencies in the dataset and to test for interactions using “treatment”, “season” (year) and their interaction as fixed effects, “month” as a repeated factor, and “replicate” as a random effect. When significant effects were detected, pairwise comparisons among treatments within each time point were performed by Tukey–HSD test. The coefficient of variation (*CV*) of fruit weight distribution for the different treatments was calculated as the ratio of the standard deviation (*SD*) to the mean for each treatment each year. Results were also subjected to a two-tailed Pearson correlation matrix using the ‘corrplot’ package in RStudio (Boston, MA), in the programming language R.

## 3 Results

### 3.1 Meteorological data in the experimental plot

**Figure 1:**
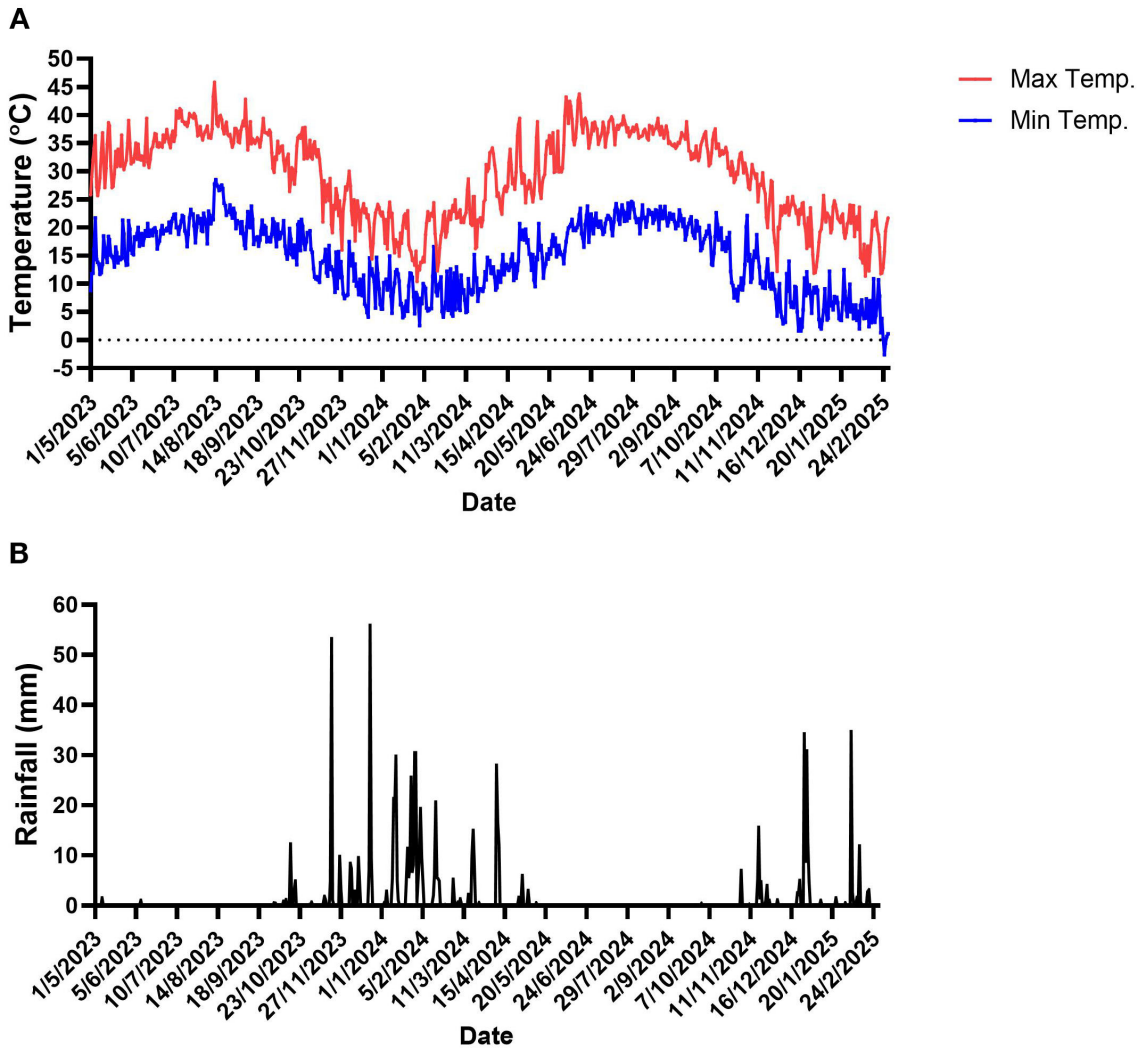
Meteorological data in the experimental plot. **(A)** Daily minimum and maximum air temperatures. **(B)** Daily rainfall. Data were recorded from May 2023 to March 2025 at `Kfar Blum` meteorological station, close to the experimental site. Dates are given as day/month/year.

During the experiment, extreme weather events occurred in the experimental orchard. Extremely high air temperatures, exceeding 40°C, occurred mostly during the summer (July and August) of 2023 and spring (June) of 2024. The highest air temperature was observed in August 2023—45.8°C. At the other end of the scale, during the winter, the air temperature dropped below 4°C in February 2024 and December 2024–February 2025 (**Figure 1A**). The lowest air temperature was observed in February 2025, -2.6 °C. Most precipitation events occurred between November and March, with several events exceeding 40mm per day. In contrast, the spring and summer months were characterized by minimal to no rainfall, consistent with a typical Mediterranean climate (**Figure 1B**).

### 3.2 Effect of UNI on trunk diameter

**Figure 2:**
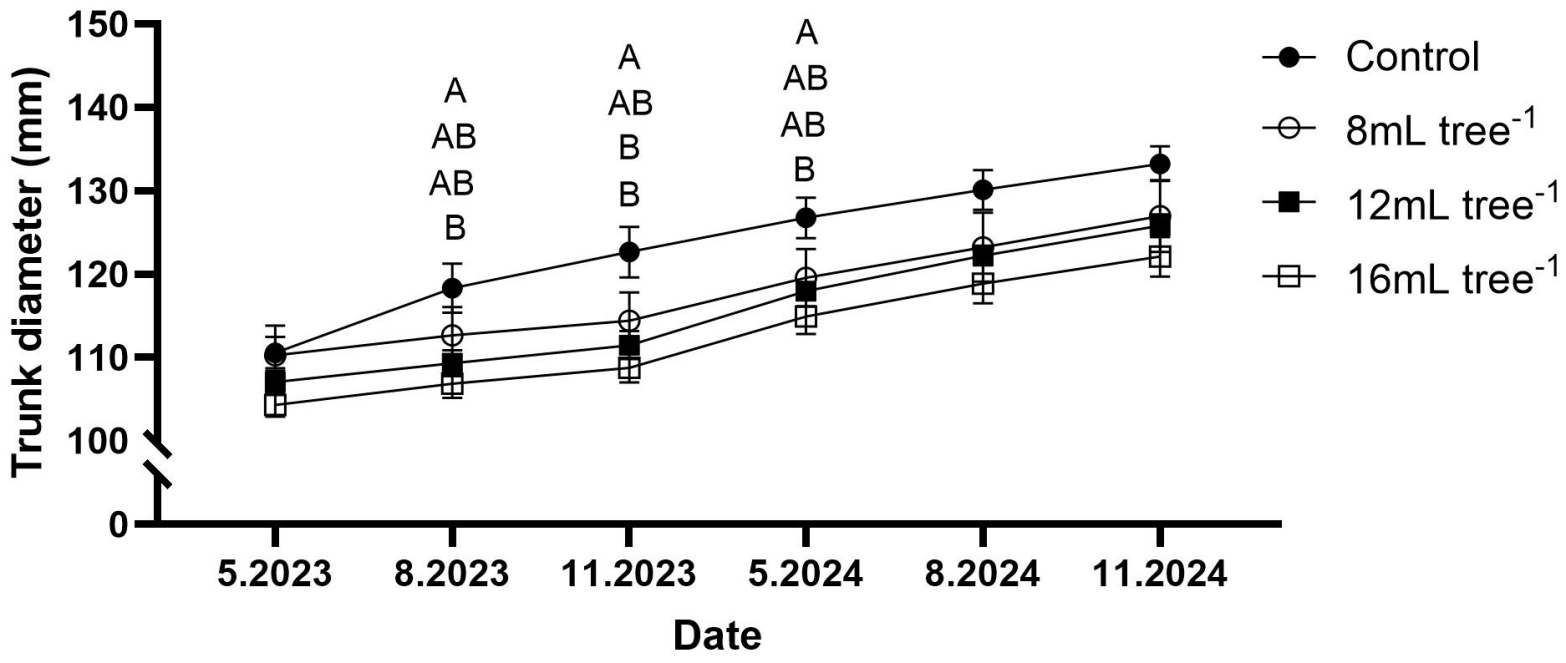
Trunk diameter growth in control and UNI-treated trees. Trunk diameter was measured at six time points: May 2023, August 2023, November 2023, May 2024, August 2024, and November 2024. Values are means ± SE of four replicates (n = 4), each comprised of eight different trees. Different letters at a given time point indicate significant difference (Tukey-HSD, *P* < 0.05).

Trunk diameter increased steadily across all treatments from May 2023 to November 2024 (**Figure 2**). However, the magnitude of growth varied with applied treatment. By August 2023, trees in the control group exhibited a significantly greater trunk diameter than the 16 mL tree^-^¹ treatment (*P* < 0.05), with intermediate values observed for the 8 mL and 12 mL tree^-1^ treatments. This pattern generally persisted through the subsequent sampling dates. At each measurement point, the control group maintained the largest trunk diameter, whereas the 16 mL tree^-1^ treatment consistently showed the lowest values. By November 2024, the trunk diameter of the control group had reached approximately 133 mm, compared to 127 mm, 126 mm, and 122 mm in the 8 mL, 12 mL, and 16 mL tree^-1^ treatments, respectively.

### 3.3 Effect of UNI on flowering intensity and inflorescence bud density

**Figure 3:**
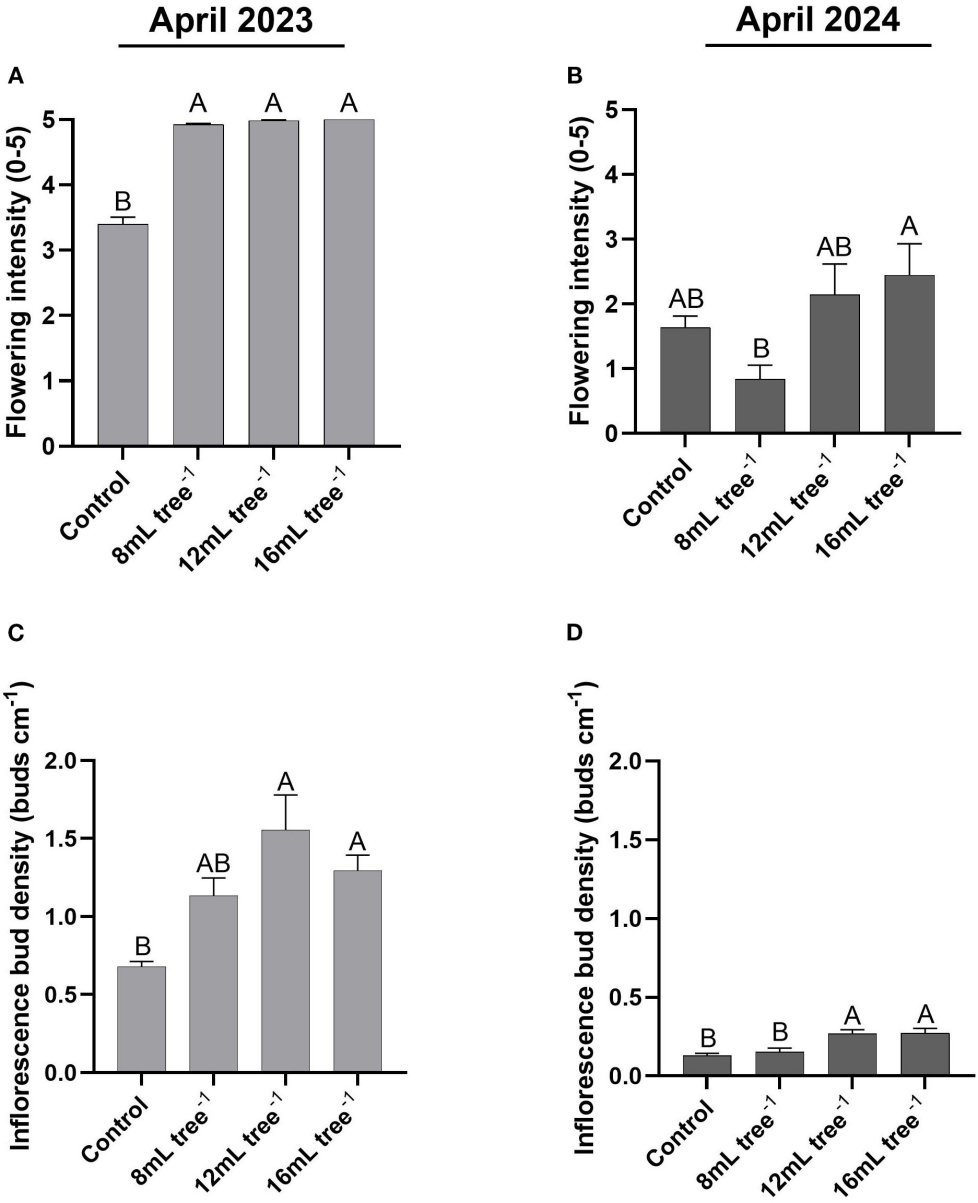
Flowering intensity and inflorescence bud density in control and UNI-treated trees. Flowering intensity in the control and UNI-treated trees was assessed and scored in April 2023 **(A)** and April 2024 **(B)** on a scale of 0–5, with 0 representing no apparent flowering and 5, maximum bloom. Inflorescence bud density was determined in April 2023 **(C)** and April 2024 **(D)** by measuring and calculating the number of floral buds along a 20-cm segment at the tip of a flowering branch. Values are means ± SE of four replicates (n = 4), each comprised of eight different trees. At least three different branches were measured from each tree. Different letters indicate significant difference between treatments within a given year (Tukey-HSD, *P* < 0.05).

In April 2023, flowering intensity was significantly affected by treatment (**Figure 3A**). All UNI treatments (8 mL, 12 mL, and 16 mL tree^-^¹) showed significantly (*P* < 0.05) higher flowering intensity scores (4.9–5.0 on a 0–5 scale) compared to the control (3.4). No significant differences were observed among the three application rates. In contrast, in April 2024, flowering intensity declined across all treatments (**Figure 3B**). The 16 mL tree^-^¹ treatment maintained the highest mean flowering intensity (2.4), significantly (*P* < 0.05) higher than the 8 mL tree^-^¹ treatment (0.8), which resulted in the lowest value. The control and 12 mL tree^-^¹ treatments exhibited intermediate values with no significant differences compared to either extreme.

A similar pattern was observed for inflorescence bud density. In April 2023 (**Figure 3C**), the control group exhibited the lowest bud density (0.68 buds cm^-^¹), significantly (*P* < 0.05) lower than the 12 mL and 16 mL tree^-^¹ treatments (1.3–1.55 buds cm^-^¹). The 8 mL tree^-^¹ treatment showed intermediate values (1.13 buds cm^-^¹) that were not significantly (*P* > 0.05) different from either the control or the higher application rates. In April 2024 (**Figure 3D**), inflorescence bud density declined markedly in all treatments. However, trees from the 12 and 16 mL tree^-^¹ treatments maintained significantly (*P* < 0.05) higher bud densities (0.27 buds cm^-^¹) than the control and 8 mL tree^-^¹ treatments (0.13–0.15 buds cm^-^¹).

### 3.4 Effect of UNI on chlorophyll content and gas-exchange measurements

**Figure 4:**
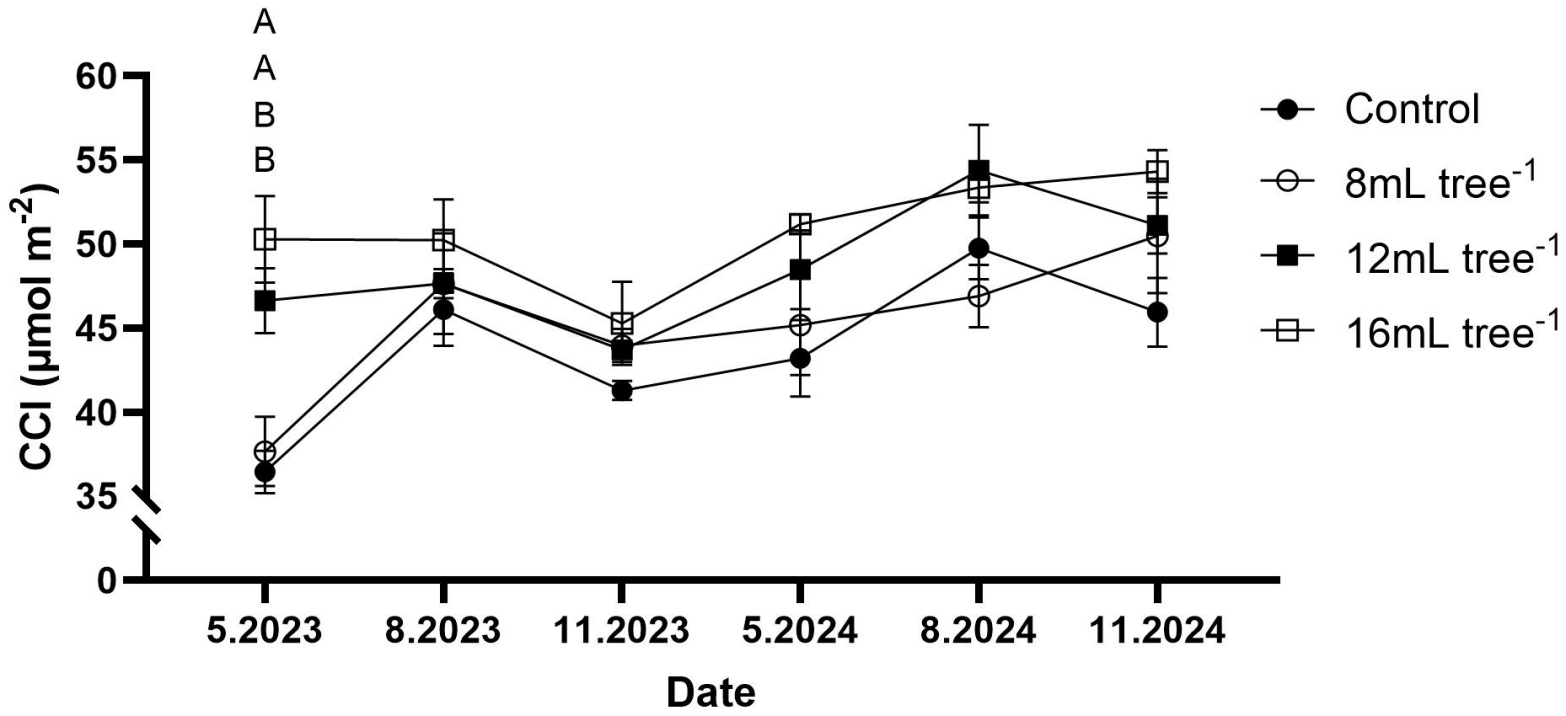
Chlorophyll content in control and UNI-treated trees. Leaf chlorophyll content (CCI) was measured at six time points: May 2023, August 2023, November 2023, May 2024, August 2024, and November 2024. Values are means ± SE of four replicates (n = 4), each comprised of eight different trees. At least three different leaves were measured from each tree. Different letters at a given time point indicate significant difference (Tukey-HSD, *P* < 0.05).

Throughout the study period, CCI exhibited seasonal fluctuations but remained higher in the 12 mL and 16 mL tree^-^¹ treatments than in controls (**Figure 4**). However, statistically significant differences (*P* < 0.05) were observed only in May 2023, when CCI values in the 12 mL and 16 mL tree^-^¹ treatments were significantly higher than those in both the control and 8 mL tree^-^¹ treatments.

**Figure 5:**
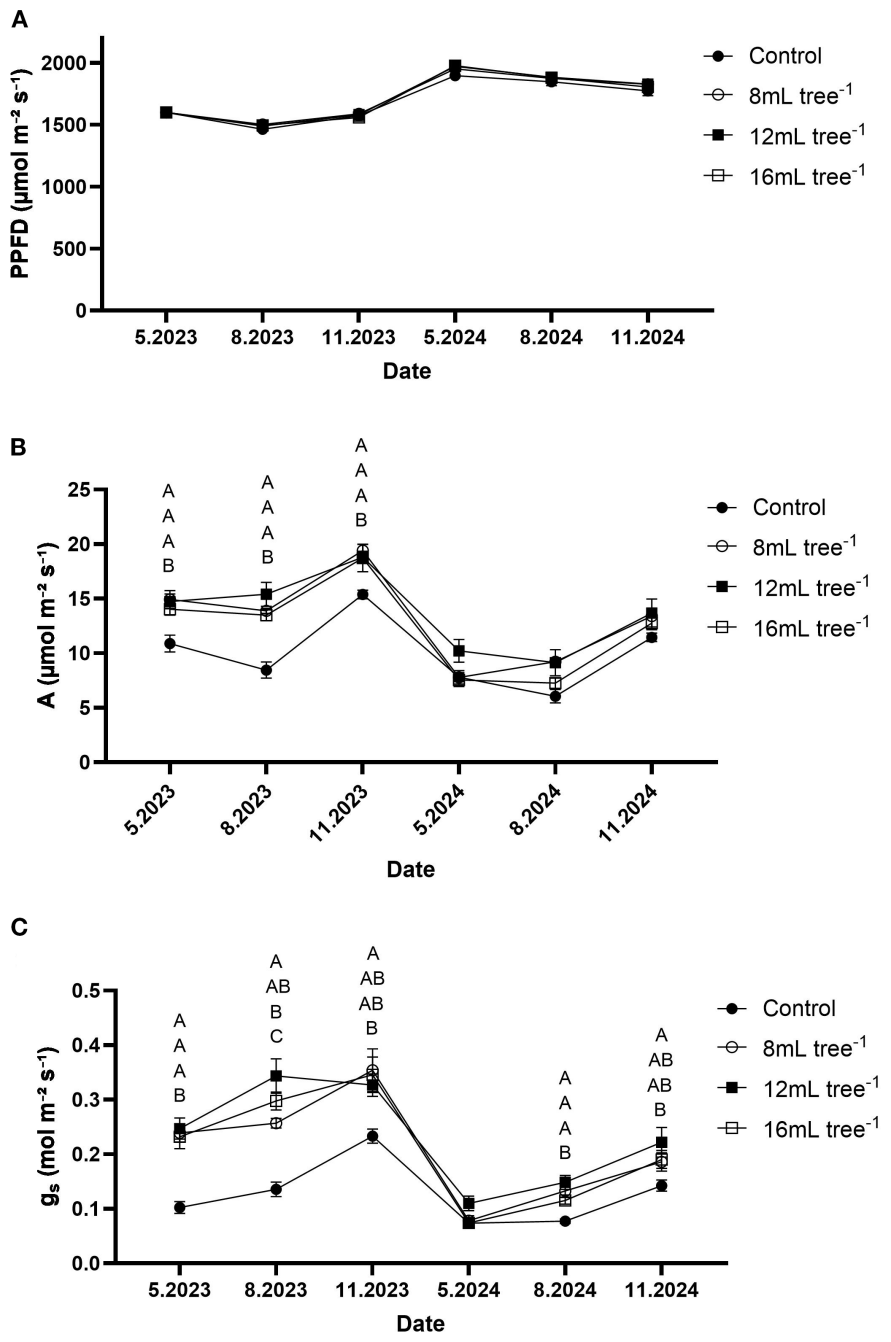
Gas-exchange parameters in control and UNI-treated trees. Leaf-level light intensity, CO_2_ assimilation and stomatal conductance were measured and calculated by the LI-COR system. **(A)** PPFD – photosynthetic photon flux density. **(B)** A – CO_2_ assimilation. **(C)** g_s_ – stomatal conductance to water vapor. Parameters were measured mid-day at six time points: May 2023, August 2023, November 2023, May 2024, August 2024, and November 2024. Values are means ± SE of four replicates (n = 4), each comprised of eight different trees. At least three different leaves were measured from each tree. Different letters at a given time point indicate significant difference (Tukey-HSD, *P* < 0.05).

PPFD values remained relatively stable throughout the study period and did not differ significantly (*P* > 0.05) among treatments (**Figure 5A**). Across all sampling dates, PPFD ranged between approximately 1,460 and 1,980 µmol m^-^² s^-^¹, indicating consistent light availability during the gas-exchange measurements. Carbon-assimilation rate showed yearly and treatment-related variations (**Figure 5B**). In general, it was higher in 2023 than in 2024. In May, August, and November 2023, trees treated with 8, 12 or 16 mL tree^-^¹ exhibited significantly (*P* < 0.05) higher carbon-assimilation rates than the control. In contrast, during 2024, there were no significant (*P* > 0.05) differences in carbon-assimilation rates between UNI-treated and control trees, but the control trees still exhibited consistently lower carbon-assimilation rates than the UNI-treated trees.

Stomatal conductance (g_s_) followed a pattern similar to that of carbon-assimilation rate and was generally higher in 2023 than in 2024. (**Figure 5C**). Although g_s_ values declined sharply in May 2024 across all treatments, moderate recovery was observed by November 2024. Across the study period, stomatal conductance was consistently enhanced by the UNI treatments compared to the control. At most sampling points, the control trees showed significantly (*P* < 0.05) lower g_s_ values than the UNI-treated trees. Moreover, at most sampling points, no significant differences were observed in g_s_ values among the UNI-treated trees. The only exception was in August 2023, when the 12 mL tree^-^¹ treatment exhibited significantly (*P* < 0.05) higher g_s_ values than the 8 mL tree^-^¹ treatment.

### 3.5 Effect of UNI on yield, average fruit weight, and size-class distribution

**Figure 6:**
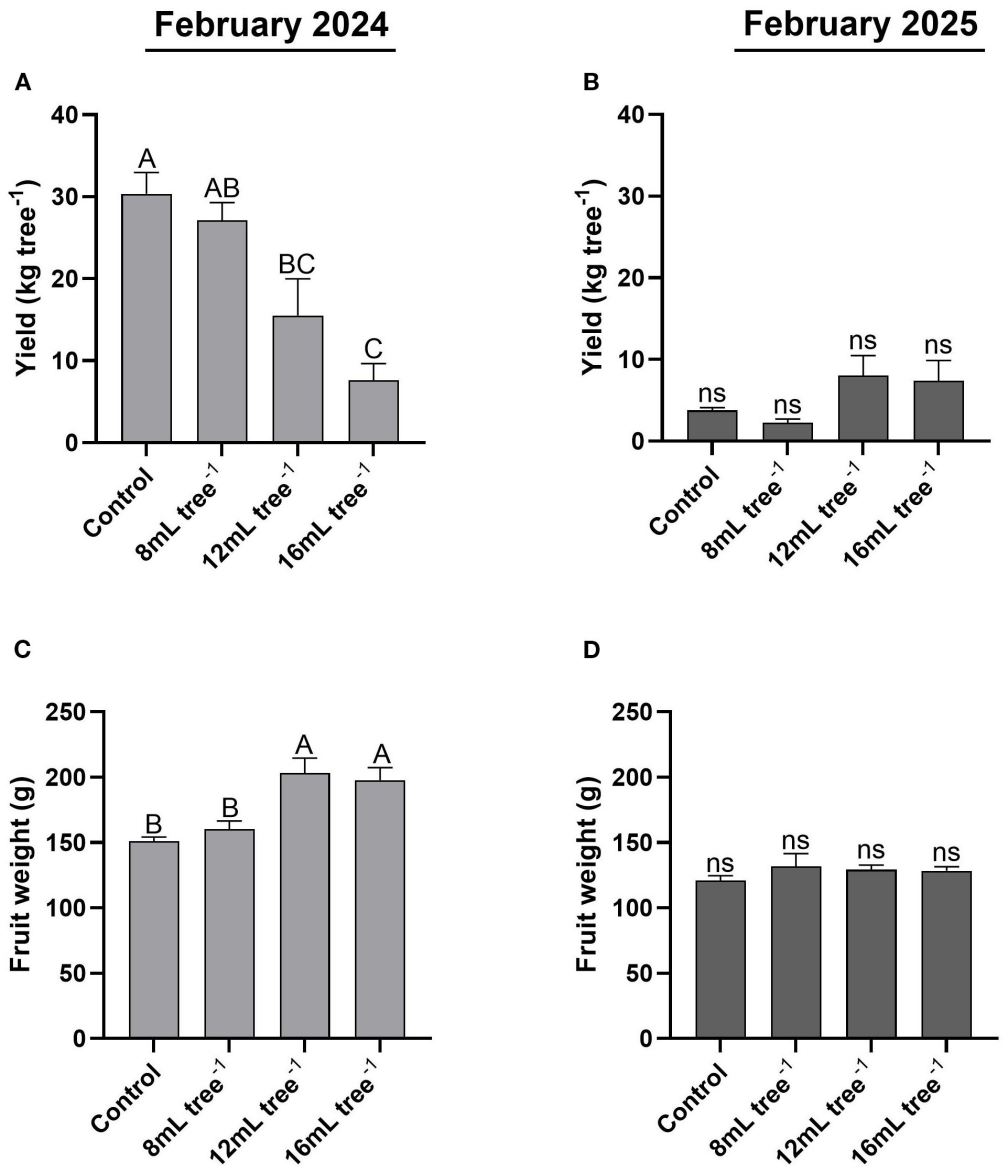
Fruit yield and average fruit weight in control and UNI-treated trees. Fruit yield in the control and UNI-treated trees was determined in February 2024 **(A)** and February 2025 **(B)**. Average fruit weight was determined in February 2024 **(C)** and February 2025 **(D)** by dividing the total fruit weight by the number of fruits on each tree. Values are means ± SE of four replicates (n = 4), each comprised of eight different trees. Different letters indicate significant difference between treatments within a given year (Tukey-HSD, *P* < 0.05); “ns” denotes non-significant.

In February 2024, the control trees had the highest yield (30 kg tree^-^¹), which was significantly (*P* < 0.05) greater than that of the 12 mL and 16 mL tree^-^¹ treatments (**Figure 6A**). The yield of the 8 mL tree^-^¹ treatment was slightly lower than the control but was significantly (*P* < 0.05) higher than that of the 16 mL tree^-^¹ treatment. In February 2025, overall yield was markedly reduced across all treatments (**Figure 6B**), with not statistically significant (*P* > 0.05) differences between them. Yields ranged from 2.3 to 8 kg tree^-^¹, reflecting a substantial decline compared to the previous year. In February 2024, average fruit weight in the 12 mL and 16 mL tree^-^¹ treatments was 203 and 198 g, respectively, with no significant (*P* > 0.05) difference between them (**Figure 6C**). In contrast, fruit weights in the control (151 g) and 8 mL tree^-^¹ (160 g) treatments were significantly (*P* < 0.05) lower than those observed at the higher UNI-application rates. In February 2025, average fruit weight was generally lower than that in 2024 and was similar across all treatments, ranging between 121 and 132 g with no significant differences between treatments (**Figure 6D**).

**Figure 7:**
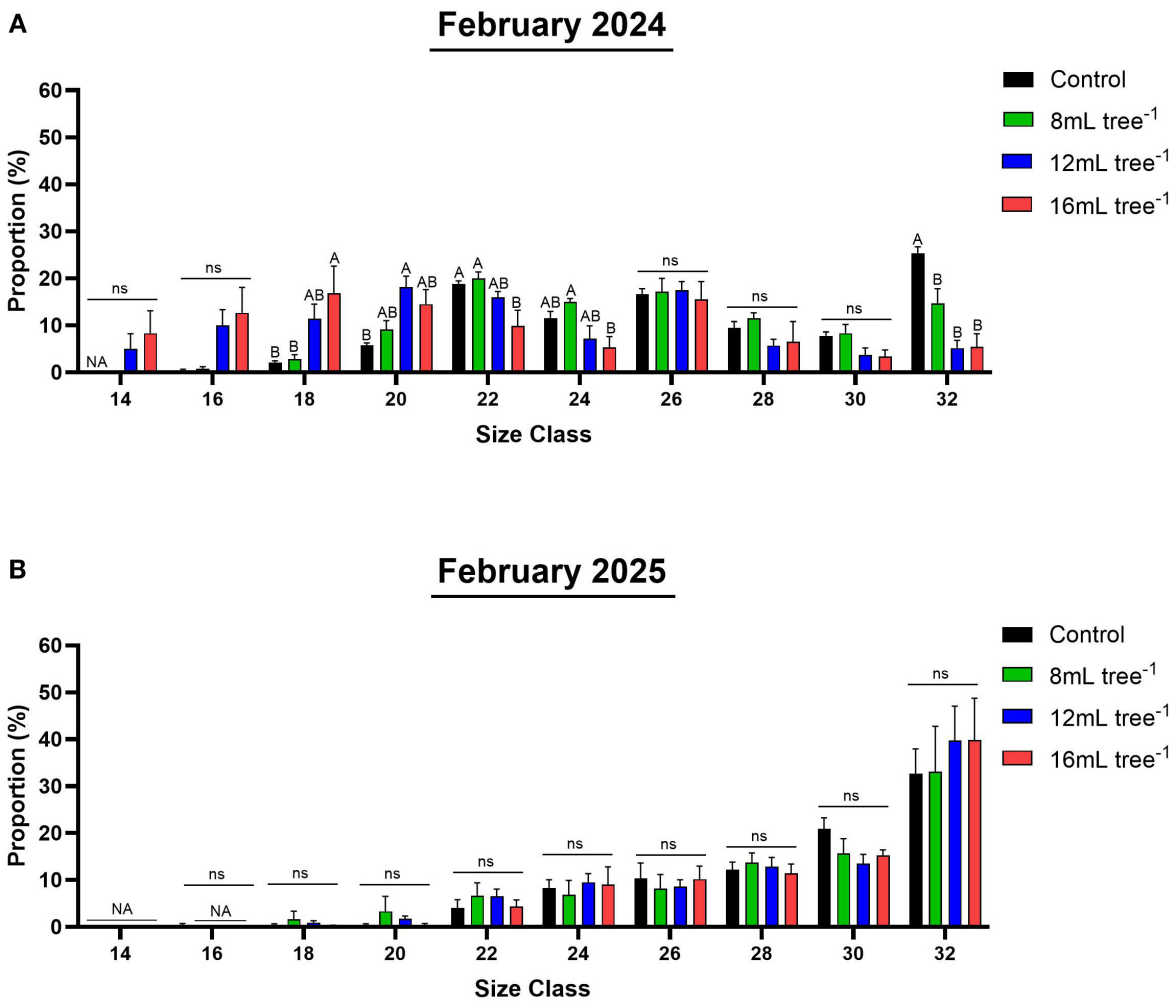
Size class distribution in control and UNI-treated trees. Individual fruit weight in representative control and UNI-treated trees was determined in February 2024 **(A)** and February 2025 **(B)** and classified by predefined commercial size classes: size 14 (266-305g), size 16 (236-265g), size 18 (211-235g), size 20 (191-210g), size 22 (171-190g), size 24 (156-170g), size 26 (146-155g), size 28 (136-145g), size 30 (125-135g) and 32 (80-125g). Values are means ± SE of four replicates (n = 4), each comprised of two different trees. Different letters indicate significant difference between treatments within a given size class (Tukey-HSD, *P* < 0.05); “ns” denotes non-significant.

Size-class distribution varied among treatments in February 2024 (**Figure 7A**; **Supplementary Table S1**). The control trees produced predominantly smaller fruit, with the highest proportion (25%) falling into the smallest 32 size class. Fruit from the 8 mL tree^-^¹ treatment showed a similar distribution, but with a significantly (*P* < 0.05) smaller proportion in the 32 size class (15%). In contrast, the 12 mL and 16 mL tree^-^¹ treatments produced a more even distribution across size classes, with a marked tendency toward heavier grades, indicating a clear treatment effect on fruit-size enhancement. In February 2025 (**Figure 7B**; **Supplementary Table S1**), size-class distributions were similar among all treatments. Notably, in all treatments, the highest proportion of fruit fell into the 32 size class.

### 3.6 Correlation between measured physiological, morphological, and yield-related parameters

**Figure 8:**
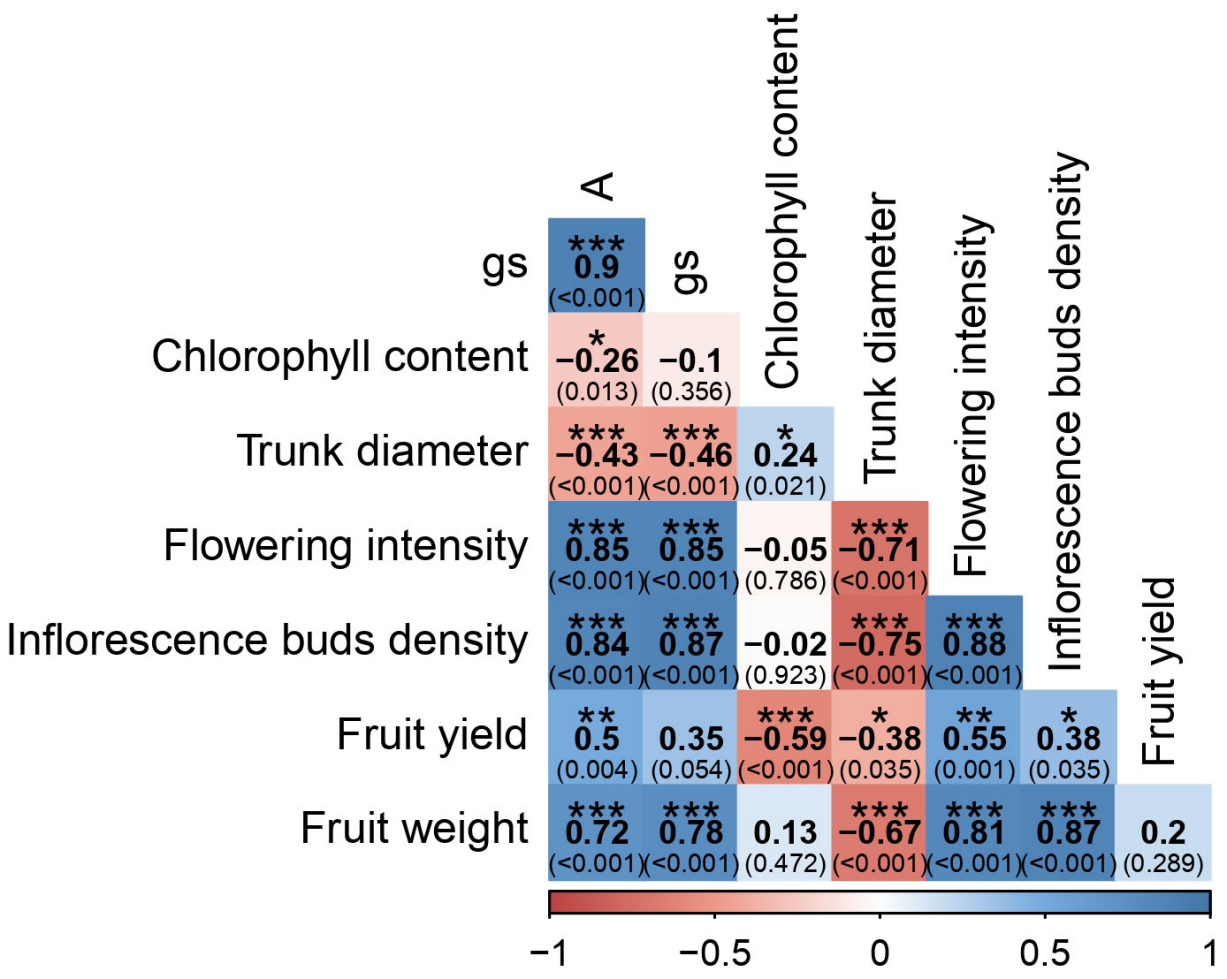
Pearson correlation coefficients among A – CO_2_ assimilation, g_s_ – stomatal conductance to water vapor, leaf chlorophyll content, trunk diameter, flowering intensity, inflorescence bud density, fruit yield and average fruit weight. Colors reflect correlation values between every two variables. Blue and red colors represent positive and negative correlations, respectively. *P*-values are shown in parentheses, and asterisks indicate significance of Pearson correlation values (*P < 0.05, **P < 0.01, ***P < 0.001) within each box. Each value represents measurements for all four treatments during the 2 years of the experiment.

Of the 28 correlation coefficients evaluated, 22 exhibited significant values (*P* < 0.05; **Figure 8**). Carbon-assimilation rate (A) was strongly and positively correlated with stomatal conductance (*g_s_*) (*r* = 0.90, *P* = 0.001), flowering intensity (*r* = 0.85, *P* < 0.001), inflorescence bud density (*r* = 0.84, *P* < 0.001), and fruit weight (*r* = 0.72, *P* < 0.001). It showed a moderate positive correlation with fruit yield (*r* = 0.50, *P* = 0.004). Conversely, A was negatively correlated with trunk diameter (*r* = −0.43, *P* < 0.001) and chlorophyll content (*r* = −0.26, *P* = 0.013). Stomatal conductance exhibited a similar pattern, being strongly positively correlated with flowering intensity (*r* = 0.85, *P* < 0.001), bud density (*r* = 0.87, *P* < 0.001), and fruit weight (*r* = 0.78, *P* < 0.001), while negatively correlated with trunk diameter (*r* = −0.46, *P* < 0.001). Flowering intensity and bud density were strongly and positively correlated (*r* = 0.88, *P* < 0.001). Both parameters were positively correlated with fruit weight (flowering: *r* = 0.81, *P* < 0.001; bud density: *r* = 0.87, *P* < 0.001) and with yield (flowering: *r* = 0.55, *P* = 0.001; bud density: *r* = 0.38, *P* = 0.035). Trunk diameter was negatively correlated with flowering intensity (r = −0.71, P < 0.001), bud density (r = −0.75, P < 0.001), fruit weight (r = −0.67, P < 0.001), and yield (r = −0.38, P = 0.035). Chlorophyll content showed a negative correlation with yield (*r* = −0.59, *P* < 0.001) and a positive correlation with trunk diameter (r = 0.24, P < 0.021). The linear mixed model analysis (**Supplementary Table S2**) confirmed that both treatment and season (year) exerted highly significant effects on all measured parameters. Treatment-by-season interactions were generally less pronounced, although significant effects were detected for g_s_, flower intensity, inflorescence bud density, fruit yield, and fruit weight.

## 4 Discussion

This study offers comprehensive insights into the effects of soil-applied UNI on vegetative growth, flowering, and physiological responses of commercial field-grown ‘Hass’ avocado trees under a Mediterranean climate. During the experimental period, the orchard experienced multiple extreme weather events, including heat waves with maximum air temperatures exceeding 40°C (peaking at 45.8°C in August 2023), and cold spells with minimum temperatures dropping below 4°C in winter, reaching -2.6°C in February 2025. These fluctuations in temperature, typical of the region’s Mediterranean climate, imposed potential environmental stresses that could negatively impact tree physiology, yield, and fruit quality (Shapira et al., 2021; Alon et al., 2022; Lahak et al., 2024), may have contributed to the interannual variability observed in yield and gas-exchange measurements, and may have modulated the effects of UNI treatments. UNI blocks cytochrome P450-dependent monooxygenases, thereby inhibiting the oxidation of *ent*-kaurene into *ent*-kauronic acid in the GA biosynthesis pathway, reducing endogenous GA levels (Rademacher, 2000). This suppression limits cell elongation in vegetative shoots, resulting in reduced trunk growth and potentially increasing the proportion of assimilates available for reproductive development (Katz et al., 2003). Compared to untreated controls, UNI-treated trees exhibited reductions in trunk diameter and an increase in floral bud density (**Figures 2**, **3**), reflecting a clear suppression of tree vigor (Reddy et al., 2003; Chernoivanov et al., 2022; Lahak et al., 2024). These outcomes are consistent with prior research demonstrating the growth-inhibitory effects of UNI. For example, foliar application of UNI markedly curtailed shoot elongation in greenhouse-grown avocado seedlings, with the extent of inhibition increasing with application rate (Köhne and Kremer-Köhne, 1989). A comparable reduction in shoot growth following UNI foliar treatment was observed in non-irrigated ‘Hass’ avocado trees (Brogio et al., 2018). UNI soil drenching led to decreased shoot length and trunk cross-sectional area in ‘Hass’ avocado trees grafted on Mexicola rootstock 9 months after container establishment, relative to their untreated counterparts (Bórquez-Lillo et al., 2015). In avocado, as well as in other species, it is desirable to control vegetative growth and reduce canopy size in commercial plantations (Mitra, 2018). Effective vegetative control is particularly advantageous in high-density orchards and to reduce labor-intensive management practices (Kohne and Kremer-Kohne, 1990; Köhne and Kremer-Köhne, 1992; Menzel and Le Lagadec, 2014). It may also prove advantageous in the context of dual-use agricultural systems integrating over-canopy solar panels, as it can help restrain excessive vegetative growth of the trees under the panels (Trommsdorff et al., 2022). However, caution is required to avoid long-term oversuppression, which could negatively impact the source–sink balance and productivity.

The results of this study demonstrate a clear promotive effect of UNI treatment on flowering during the first season, with this effect partially persisting into the second season despite an overall reduction in reproductive activity (**Figure 3**). Flowering intensity was negatively correlated with trunk diameter (**Figure 8**), suggesting that trees allocating more resources to vegetative structural growth may do so at the expense of reproductive development. Such a trade-off between vegetative vigor and reproductive growth is well-documented in avocado (Wolstenholme et al., 1990), and may be influenced by hormonal regulation, particularly gibberellin-mediated growth promotion at the expense of floral initiation. Similar findings have been reported in other studies; for instance, UNI soil applications increased both the proportion of floral to total buds and overall flowering intensity in young ‘Hass’ avocado trees (Bórquez-Lillo et al., 2015). In litchi, enhanced flowering was observed with PBZ treatments only when vegetative growth was suppressed for 1 to 2 months before panicle emergence (Menzel and Simpson, 1990). Similar responses have also been documented in mango and citrus (Mitra, 2018). It has been suggested that inhibition of GA biosynthesis, particularly under conditions of reduced photosynthetic activity, limits vegetative growth and reallocates assimilates toward the shoot apex, thereby promoting floral induction (Katz et al., 2003). In addition, the export of GAs from developing seeds to nearby buds has been implicated in floral suppression in several fruit tree species, including avocado (Guardiola et al., 1982; Salazar-García and Lovatt, 2000). Indeed, canopy spray application of GA_3_ at concentrations of 25 or 100 mg L^-^¹ in November, prior to the “on” bloom, resulted in a reduced number of inflorescences, an increase in vegetative shoot production, and a 47% decrease in yield compared to untreated control trees (Salazar-García and Lovatt, 2000). Moreover, research in mango and citrus suggests that GA may inhibit flowering by downregulating the accumulation of the *FLOWERING LOCUS T* (*FT*)-like gene, hypothesized to act as a phloem-mobile florigen signal encoding mRNA accumulation in both leaves and buds (Nakagawa et al., 2012; Goldberg-Moeller et al., 2013; Ziv et al., 2014; De Sousa Lima et al., 2016). In the shoot meristem, FT interacts with the bZIP transcription factor FD to promote the floral transition by inducing the expression of MADS-box genes, including *SUPPRESSOR OF OVEREXPRESSION OF CONSTANS1* (*SOC1*), *FRUITFULL* (*FUL*) and *APETALA1* (*AP1*). These MADS-box transcription factors, in turn, activate floral meristem identity genes such as *LEAFY* (*LFY*), which encodes a unique plant transcription factor essential for flower development (Mouradov et al., 2002; Bäurle and Dean, 2006; Ziv et al., 2014). These floral molecular pathways were not examined in the present study; future research should incorporate molecular analyses to enhance the mechanistic understanding of the UNI treatments.

Physiological measures demonstrated that UNI-treated trees generally maintain higher chlorophyll content and enhanced rates of carbon assimilation and stomatal conductance compared to controls during the first year (**Figures 4**, **5**). These effects were less pronounced in the second year, likely due to the reduced persistence of the UNI effects on the trees. Although carbon assimilation and stomatal conductance were slightly negatively correlated with chlorophyll content (**Figure 8**), the increased photosynthetic efficiency can be related to the increase in leaf chlorophyll content (Lahak et al., 2024). In longan, application of UNI increased leaf chlorophyll contents and net photosynthetic rate (Nie et al., 2001). In mango, soil application of PBZ also increased leaf chlorophyll content (Mitra, 2018), and enhanced chlorophyll content and carbon-assimilation rates were observed in oil palms treated with PBZ (Chai et al., 2023). Notably, transcriptome analysis in the latter study showed that PBZ treatment upregulates the expression of *geranylgeranyl diphosphate reductase*, a key enzyme involved in chlorophyll biosynthesis. In contrast, GA_3_ application downregulated genes associated with chlorophyll synthesis, as well as those encoding components and assembly factors of the photosystem I and II super complexes and Rubisco (Chai et al., 2023). In water-stressed soybean plants, foliar application of uniconazole resulted in higher chlorophyll content and photosynthetic rates compared to untreated controls. This effect was attributed to UNI-induced enhancement of antioxidant enzyme activities, increased accumulation of proline and soluble sugars, and reduced levels of malondialdehyde (MDA), collectively contributing to improved membrane stability and sustained photosynthetic performance under stress conditions (Zhang et al., 2007). The mechanistic pathways by which UNI enhances leaf chlorophyll content and photosynthetic rate in avocado warrant further investigation. Still, the increased carbon-assimilation rates observed in our study may offer added value for carbon credit systems, enabling avocado producers to generate additional income by selling carbon credits to industries or producers with higher greenhouse gas emissions (Lokuge and Anders, 2022; Trouwloon et al., 2023). The positive correlations observed between carbon assimilation, stomatal conductance, flowering intensity, and fruit weight (**Figure 8**) suggest that photosynthetic performance is a central driver of both reproductive potential and fruit development in avocado trees under the conditions of this study.

Although flowering intensity in the first year of the 12 and 16 mL tree^-1^ treatments was higher than the control, fruit yield was significantly lower (**Figures 3**, **6**). In the second year, yields were notably low in both the control and 8 mL tree^-^¹ treatments, likely reflecting an alternate bearing pattern (Lovatt, 2010; Pochamreddy et al., 2024). Although the 12 and 16 mL tree^-^¹ treatments were expected to enter an “on” year with higher yields, their production remained low, indicating a potential suppressive effect of UNI on yield. This decoupling of flowering and yield corroborates reports of high floral intensity not necessarily translating to greater yield (Rubinovich et al., 2024). Still, data from both years show a positive correlation between flowering intensity and fruit yield (**Figure 8**). The lower yields in the UNI-treated trees may result from reduced carbohydrate reserves due to suppressed vegetative vigor, increased fruitlet abscission, or heightened sensitivity to pathogens such as *Botryosphaeria* under the adverse environmental conditions of the experimental plot (**Figure 1**), which may contribute to branch dieback and, consequently, increased fruitlet drop in these treatments (Valencia et al., 2019). These parameters warrant investigation in future studies. These findings contrast with those of Adato (Adato, 1990), who reported that early spring spraying of avocado cv. Fuerte increases yields. Similarly, mango trees treated with PBZ consistently outperformed untreated trees, with average 5-year yield of 123 kg in treated trees compared to 91 kg in controls (Mitra, 2018). Here, in the first year, the 12 and 16 mL tree^–1^ treatment resulted in significantly higher average fruit weight compared to the control (**Figure 6**). Moreover, these treatments exhibited a more uniform distribution across weight categories, with a noticeable shift toward heavier fruit grades, highlighting a clear effect of the treatment on enhancing fruit size (**Figure 7**). This outcome aligns with the commonly observed negative correlation between fruit weight and yield (Mickelbart et al., 2012). In addition, Stern et al. (2009) demonstrated that applying UNI at 30% full bloom in cherry trees leads to a reduction in fruit-set percentage, and therefore a significant decrease in crop load, resulting in a higher proportion of larger fruit. Given the strong economic dependence of avocado prices on fruit size, the commercial advantage of such treatment-induced size enhancement could potentially offset yield reductions, contingent on market conditions. Thus, the impact of UNI treatments on yield components warrants careful consideration, and a financial analysis should be conducted to assess their overall commercial viability.

The results from this study showed significant effects of both treatment and season (year) on physiological and yield-related parameters (**Supplementary Table S2**). This indicates that treatments had consistent effects across years, despite considerable seasonal variability. Significant interactions for g_s_, flowering intensity, inflorescence bud density, fruit yield, and fruit weight indicate that the magnitude of UNI responses varied between years, possibly reflecting seasonal climate effects and alternate bearing. Although this study sheds light on the phenological and physiological effects of UNI on avocado trees, the molecular and physiological mechanisms of this growth retardant in avocado remain unclear. It should be noted that the lack of measurements on soil nutrient availability and UNI mobility and degradation rates limits our ability to fully explain the physiological responses observed. Given that soil texture and chemistry can influence the mobility and persistence of triazole growth retardants (Deng et al., 2012; Menzel and Le Lagadec, 2014), future studies should integrate molecular and physiological investigation and soil monitoring to improve mechanistic understanding.

## 5 Conclusion

This study demonstrates that soil application of UNI is an effective tool for managing vegetative vigor and modulating reproductive development in mature ‘Hass’ avocado trees under a Mediterranean climate. Taking together, the findings support the strategic use of UNI to optimize canopy structure and fruit quality in commercial avocado orchards. However, the observed trade-offs between vegetative suppression, yield quantity, and fruit size highlight the need for precise dose calibration and long-term evaluation. To aid decision-making, we present a comparative summary of the main advantages and disadvantages of each treatment (**Supplementary Table S3**). The 8 mL tree^-^¹ treatment provided a balanced outcome- moderate vegetative control, improved physiological performance, and partially enhanced fruit size- while maintaining relatively high yields. The 12 and 16 mL tree^-^¹ treatments produced larger fruit and improved size distribution in the first year, but were associated with yield reductions, especially in the second year. The untreated control maintained the highest yield in the first year but produced the smallest fruit with poor size-class distribution and no vegetative growth control. Overall, the 8 mL tree^-^¹ treatment can be considered the most balanced approach within the scope of this study, particularly when both yield and fruit size are economically relevant. Future studies should further investigate the long-term cumulative effects of repeated UNI applications on tree architecture, photosynthetic capacity, and yield sustainability, as well as their integration into high-density planting systems and climate resilience strategies.

## Data Availability

The original contributions presented in the study are included in the article/**Supplementary Material**. Further inquiries can be directed to the corresponding author.

## Supplementary material

Supplementary Table 1
*CV* of fruit weight distribution for the different treatments in 2024 and 2025.

Supplementary Table 2 Summary of fixed effects tests from linear mixed model analyses of treatment, season (year), and their interaction on physiological and agronomic traits in the experimental trees.

Supplementary Table 3 Summary of the main advantages and disadvantages of control and UNI treatments at different application rates (8, 12, and 16 mL tree^-^¹) in mature ‘Hass’ avocado trees.
