# Supplementary material for: Effects of uniconazole treatment on ‘Hass’ avocado productivity and gas-exchange parameters under Mediterranean climate
Source: Front Plant Sci. 2025 Sep 19;16:1668625. doi: 10.3389/fpls.2025.1668625 (PMC12491040; doi:10.3389/fpls.2025.1668625)
Supplement: Supplementary Table 1 — CV of fruit weight distribution for the different treatments in 2024 and 2025. [file Table1.docx]

**Supplementary Table S1.** *CV* of fruit weight distribution for the different treatments in 2024 and 2025.

| Treatment | **2024** | **2025** |
| --- | --- | --- |
| Control | 23.98 | 25.27 |
| 8mL tree^-1^ | 20.25 | 25.00 |
| 12mL tree^-1^ | 20.71 | 25.00 |
| 16mL tree^-1^ | 24.61 | 25.62 |
