# Supplementary material for: Effects of uniconazole treatment on ‘Hass’ avocado productivity and gas-exchange parameters under Mediterranean climate
Source: Front Plant Sci. 2025 Sep 19;16:1668625. doi: 10.3389/fpls.2025.1668625 (PMC12491040; doi:10.3389/fpls.2025.1668625)
Supplement: Supplementary Table 2 — Summary of fixed effects tests from linear mixed model analyses of treatment, season (year), and their interaction on physiological and agronomic traits in the experimental trees. [file Table2.docx]

**Supplementary Table S2.**  Summary of fixed effects tests from linear mixed model analyses of treatment, season (year), and their interaction on physiological and agronomic traits in the experimental trees. A – CO_2_ assimilation, g_s_ – stomatal conductance to water vapor. Asterisks indicate significance at * p < 0.05, ** p < 0.01, *** p < 0.001, “ns” denotes non-significant.

|  | **A** | **g_s_** | **Chlorophyll content** | **Trunk diameter** | **Flowering intensity** | **Inflorescence buds density** | **Fruit yield** | **Fruit weight** |
| --- | --- | --- | --- | --- | --- | --- | --- | --- |
| Treatment | ** | *** | * | ** | ** | ** | ** | ** |
| Season (year) | *** | *** | *** | *** | *** | *** | *** | *** |
| Treatment*Season (year) | ns | ** | ns | ns | ** | * | ** | ** |
