# Supplementary material for: Effects of uniconazole treatment on ‘Hass’ avocado productivity and gas-exchange parameters under Mediterranean climate
Source: Front Plant Sci. 2025 Sep 19;16:1668625. doi: 10.3389/fpls.2025.1668625 (PMC12491040; doi:10.3389/fpls.2025.1668625)
Supplement: Supplementary Table 3 — Summary of the main advantages and disadvantages of control and UNI treatments at different application rates (8, 12, and 16 mL tree-¹) in mature ‘Hass’ avocado trees. [file Table3.docx]

**Supplementary Table S3.** Summary of the main advantages and disadvantages of control and UNI treatments at different application rates (8, 12, and 16 mL tree⁻¹) in mature ‘Hass’ avocado trees. The comparison integrates effects on yield, fruit size, vegetative growth control, and physiological performance, based on two years of field data.

| **Treatment** | **Advantages** | **Disadvantages** |
| --- | --- | --- |
| Control | Highest yield; no chemical cost | Smallest fruit weight; poor size-class distribution; no vegetative growth control |
| 8mL tree^-1^ | Balanced yield and vegetative control; improved carbon assimilation; moderate increase in fruit size; slight improvement in size-class distribution | Slight yield reduction compared to control; less size-class improvement than higher doses |
| 12mL tree^-1^ | Significant increase in fruit weight; improved carbon assimilation; improved size-class distribution; strong vegetative suppression | Yield reduction; possible over-suppression of vegetative growth |
| 16mL tree^-1^ | Significant increase in fruit weight; improved carbon assimilation; improved size-class distribution; strong vegetative suppression | Lowest yield; possible over-suppression of vegetative growth |
